# Regulating the Practice

**Published:** 1891-10

**Authors:** 


### "REGULATING THE PRACTICE."

If there is any one thing in connection with medical politics that has grown threadbare, stale and unprofitable, it is the cry—"regulate the practice." It is a veritable "chestnut," in the parlance of the day.

Most unfortunate was the selection of the term as the caption of the several "medical bills," so called, which have, from time to time, been introduced in the Legislature; especially, in the first place, as it is misleading, and does not convey an idea of what is really sought to be accomplished by the medical profession; secondly, because the very proposition, coming from the "regular profession," arouses antagonism, not only in homeopaths and eclectics, but in the members of the Legislature themselves. It is not conceded to the "old school" of physicians—improperly so called by nearly every body but themselves,—to "regulate" any thing; nor is it sought by them to do so; yet this unfortunate caption has created such impression. The profession is thus misunderstood,—and being misunderstood, its requests, in all other respects reasonable and proper, are refused. Refused, did we say? Repeatedly rejected by the Legislature,—ridiculed, and treated with mocking contempt! Are these things not enough to induce medical men, the conscientious and well-meaning members of the profession, to desist?

It seems not. Every now and then some one comes forward with the draft of a bill still to "regulate the practice," and insists that it is "just what we need." The JOURNAL is in receipt of a bill of the kind, carefully prepared, by a most excellent and well meaning gentleman, who asks that it be published now, in order that it may be properly considered and discussed by medical men and legislators in advance of the next session of the Legislature. We deem its publication entirely unnecessary,—it will do no good, and its length is so great we cannot give it room.

The author knows that for seven consecutive years the Texas State Medical Association has, through an able committee, presented each year some such medical bill to the Legislature, and that strong pressure has been brought to bear to secure its passage. These several committees had repeatedly been told "if the people want such a law it will be passed." Well, with the last bill presented, they got fifteen thousand (other) people, (the profession itself, numbering about four thousand "people"), to sign a petition asking for its passage. If possible, this request was worse ridiculed than any of its unfortunate predecessors.

But the Doctor does not know that during the last Legislature, three members in the House and two in the Senate, besides an excellent doctor outside the State Association, each got up a medical bill which the author thought was "just what we need," and infinitely better than that drafted by the Association's committee, who had studied all the laws on the subject in other States, and had given seven years careful thought to it. But, all these bills contained one and the same defect, vital alike to all—that unfortunate caption to "regulate the practice."

A Senator, who, impressed with the necessity of reform in the matter of practice of medicine was really desirous of aiding the committee, asked the writer, "Do you gentlemen propose to regulate the practice of homeopaths and others, not of your school?" "Surely not," we replied; "we do not seek to regulate *anything*. The object in seeking legislation is to *restrict the privilege of practicing medicine to the hands of those who can show to a legally constituted authority that they possess the necessary qualifications for its exercise, no matter how, when or where they obtained those qualifications;* to prevent the indiscriminate practice of a profession, dangerous in its very nature, and to punish unprofessional conduct." "That is a very different thing," said the Senator, "and such a measure should pass, but your bill does not so state."

Will our people never learn wisdom? Will they never learn, no matter how often rebuffed, that the Legislature does not concede to them the right to regulate anything ; and cease to frame their bills under this misleading caption ?

A bill to punish unprofessional conduct, would pass, if properly presented.—(Such a bill as is in force in Arkansas.) Another bill to prevent the practice of medicine by incompetent persons, if properly framed, would receive the cordial support of every well meaning and intelligent member.

It is in vain that they are assured that it is not "a doctor's fight;" that the "old school" (?) is not making war on the homeopaths and eclectics; they cannot be made to believe it until we cease presenting bills to "regulate the practice."

We are satisfied the homeoopaths and eclectics would not co-operate with the regular medical profession in any effort to "suppress quackery," or to punish unprofessional conduct, or to restrict the privilege of practicing to the educated, although this would not discriminate against educated persons, no matter whether educated in a regular school or in the homeopathic school, and if educated, they should be able to stand an examination: and that is the only test. They would fight anything; for it is this that keeps them alive; the more chance they get to air themselves as the "object" of our legislation, and cry persecution, the more prominence they get. They are humbugs, and they know it, and really, as we have before shown, have no right to be even considered as factors in the subject.

